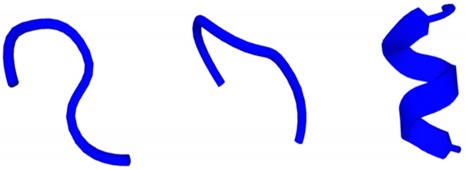# Mapping Epitopes of a Novel Peptidoglycan Cross-Linking Enzyme Cwp22 Recognized by Human Sera Obtained from Patients with *Clostridioides difficile* Infection and Cord Blood

**DOI:** 10.3390/microorganisms7110565

**Published:** 2019-11-14

**Authors:** Agnieszka Razim, Katarzyna Pacyga, Gajane Martirosian, Andrzej Szuba, Andrzej Gamian, Andrzej Myc, Sabina Górska

**Affiliations:** 1 Department of Immunology of Infectious Diseases, Hirszfeld Institute of Immunology and Experimental Therapy, Polish Academy of Sciences, 53-114 Wroclaw, Poland; andrzej.gamian@hirszfeld.pl (A.G.); myca@umich.edu (A.M.); 2 Department of Microbiology, Hirszfeld Institute of Immunology and Experimental Therapy, Polish Academy of Sciences, 53-114 Wroclaw, Poland; katarzyna.pacyga@hirszfeld.pl; 3 Department of Medical Microbiology, School of Medicine in Katowice, Medical University of Silesia, 40-752 Katowice, Poland; gmartir@sum.edu.pl; 4 Division of Angiology, Wroclaw Medical University, 51-618 Wroclaw, Poland; andrzej.szuba@umed.wroc.pl; 5 Department of Internal Medicine, 4th Military Hospital in Wroclaw, 50-981 Wroclaw, Poland; 6 MNIMBS, Department of Internal Medicine, University of Michigan, Ann Arbor, MI 48109-5648, USA

**Keywords:** vaccine, epitope, surface protein, peptide

## Abstract

*Clostridioides difficile* (CD) cause a severe diarrhea which can lead to pseudomembranous colitis and even patient death. CD infection (CDI) is connected mainly with changes in intestinal microbiota as a consequence of antibiotic treatment. The growing resistance to antibiotics, justifies the search for new methods of combating CD. Despite of ongoing research on the immunity against the pathogen, there is still lack of any reliable vaccine. Most recently, Cwp22, that is a cross-linking enzyme involved in the production of CD peptidoglycan, seems to be a promising target to prevent CDI in high-risk patients. In this paper, the Cwp22 protein polypeptide-specific epitopes were mapped in silico and using PEPSCAN procedure. They were recognized not only by antibodies from CDI patients’ but also by umbilical cord blood sera. We identified three epitopes ^54^EFRVAT^59^, ^201^KVNGKM^206^ and ^268^WQEKNGKKYY^277^ of Cwp22 protein. Since Cwp22 protein has key functionality and the described above epitopes are also recognized by umbilical cord blood serum, we postulate that they could have important protective properties. In this paper, we propose Cwp22 protein as a good antigen candidate for CDI preventive vaccine. Our results open the possibility to use ^54^EFRVAT^59^, ^201^KVNGKM^206^ and ^268^WQEKNGKKYY^277^, epitopes as suitable anti-CD vaccine antigens.

## 1. Introduction

*Clostridioides difficile* (CD), previously known as *Clostridium difficile* [1], is a Gram-positive opportunistic bacterium that is a constituent of normal gut microbiota in 3% of the adult population. The asymptomatic carriage is higher for hospitalized patients and medical personnel (10–30%) [2]. The bacteria becomes dangerous when the both qualitative and quantitative composition of gut microbiota is distressed [3]; usually as a result of the antibiotic such as clindamycin, cephalosporins or fluoroquinolones treatment [4]. The dysbiosis leads to the *C. difficile* infection (CDI). CD easily overgrows in the disrupted patient gut, starts to secrete main virulence factors, such as: toxins TcdA and TcdB. The CDI symptoms are caused by toxin-depended cytoskeleton damage of epithelial cells which leads to severe diarrhea [5], pseudomembranous colitis, or even death. The infection relapses frequently. Moreover, CD produces inexhaustible amounts of spores which are resistant to many disinfectants.

The group of the highest CDI risk patients includes the elderly, hospitalized patients and people after multiple and sustained antibiotic treatments [6]. However, recent observations of the incidence of CDI show that the risk group is expanding since the disease is affecting much younger people without antibiotic treatment and hospitalization history [7]. The only widely used CDI therapy is antibiotic treatment, in particular with fidaxomicin, vancomycin, or metronidazole [8]. Prevention is based on the isolation of confirmed cases of CDI, applying recommended hand hygiene practices and performing environmental cleaning with sporicidal agents. There are no anti-CD vaccines on the market. Nevertheless, the design of vaccines against CDI is extensively studied.

So far, the most advanced anti-CD vaccine formulations are based on CD toxins. The formalin-inactivated toxin-based, alum-adjuvanted vaccine of Sanofi Pasteur is after the third stage of clinical study (NCT02052726) [9,10]. Another vaccine under development is based on genetically modified CD toxins (Pfizer, NCT 02561195). The third one is based on a recombinant protein consisting of shortened toxins A and B amino acid sequences VLA84 (Valneva, NCT02316470). However, it was suggested that the vaccine should target also surface components of the bacteria to prevent its adhesion and colonization [11]. A vaccine composed of toxin-derived and surface antigens might be required for full protection. The non-toxoid vaccine approaches under development include using CD surface proteins like Cwp84, FliC, FliD, GroEL, and surface structures like polysaccharides and lipoteichoic acid [12].

In this study we identified a new immunoreactive protein that turned to be one of the cell wall proteins (Cwp22). Most recently, the paper about Cwp22 protein has been published characterizing its functionality [13]. Cwp22 protein is a l,d-transpeptidase, peptidoglycan cross-linking enzyme, which mutation leads to decreased toxin production at early stage of bacteria growth along with its delayed sporulation and lower motility. We mapped the amino acid sequence of Cwp22 protein and described immunoreactive epitopes that can serve as new anti-CD vaccine targets.

## 2. Materials and Methods

### 2.1. Blood Sera

#### 2.1.1. Human Peripheral Blood Sera

In this study, peripheral blood sera from patients diagnosed with CDI (*n* = 15) were collected, pooled, aliquoted, and frozen for further experiments. The diagnosis of CDI was based on more than 3 loose stools in 24 h and positive C. Diff Quik Chek Complete test results (Techlab, Blackburg, VA, USA). The sera were obtained from 4^th^ Military Hospital in Wroclaw upon written approval received from the Medical Ethics Commission of the Medical University of Wroclaw (approval No KB-631/2015, approved on 26 November 2015) and were conducted in accordance with the Helsinki Declaration, 1975. A written informed consent was obtained from each patient.

Peripheral blood sera samples from healthy volunteers (without the history of CDI) (*n* = 21) were used as healthy control. Performed experiments were approved by the Medical Ethics Committee of the Medical University of Wroclaw (approval No KB-631/2015, approved on 26 November 2015). Patients’ and healthy volunteers’ written informed consent was collected. Experiments were conducted in accordance with the Helsinki Declaration, 1975.

#### 2.1.2. Human Umbilical Cord Blood Sera

Human umbilical cord blood sera (pooled *n* = 10) used for epitope mapping were obtained from the Obstetric Clinic of the Medical University of Wroclaw (approval No KB-631/2015, approved on 26 November 2015), collected from healthy women. Samples were obtained with patients who signed the written consent.

### 2.2. Bacterial Strains

A clinical strain of *C. difficile* (CD20) was used for the immunoreactive protein identification (deposited in Polish Collection of Microorganisms as PCM2826). This strain was characterized by us previously [14]. Shortly, it produces toxins TcdA, TcdB, and binary toxin, is resistant to moxifloxacin, ciprofloxacin, erythromycin and imipenem. Also, we used a CD strain R20291 obtained from DSMZ-German Collection of Microorganisms and Cell Cultures as a pathogenic reference strain. CD was cultured anaerobically in Brain Heart Infusion Broth (BHI, Oxoid, Hempshire, UK) medium supplemented with 0.05% l-cysteine hydrochloride, at 37 °C for 48 h. Bacterial culture was spun down at 6000× *g* for 15 min and washed twice in phosphate-buffered saline (PBS) pH 7.4 before further use.

### 2.3. Protein Isolation

Surface proteins of CD were isolated using a “washing off” method employing 1 M LiCl [15]. Shortly, 1 g of bacterial mass was suspended in 7 mL of freshly prepared 1 M LiCl and incubated at room temperature (RT) for 1 h, with shaking. Then, the mixture was centrifuged at 6000× *g* for 5 min (Heraeus Contifuge Stratus, Thermo Fisher Scientific, Waltham, MA, USA). The supernatant was collected and dialyzed against water for at least 48 h with frequent water change. The protein solution was then concentrated with Amicon Ultra (Thermo Fisher Scientific, Waltham, MA, USA), MWCO = 10,000 Da. Protein concentration was estimated with Pierce™ BCA Protein Assay Kit (Thermo Fisher Scientific, Waltham, MA, USA) according to instruction provided by supplier. The protein solution was aliquoted and stored at −20 °C until used.

### 2.4. SDS-PAGE Electrophoresis

The electrophoretic profile of CD surface proteins was evaluated according to Laemmli, et al. [16] with some modifications. Briefly, two 12.5% polyacrylamide gels were used with dimensions of 18 × 16 cm, 1.5 mm thickness. The gel was resolved in SE600 Standard Dual Cooled Vertical Unit (Hoefer, Holliston, MA, USA). The electrophoresis buffer used consisted of 0.025 M Tris, 0.192 M glycine and 0.1% sodium dodecyl sulphate (SDS). Before the electrophoresis, protein samples were suspended in Laemmli buffer and denatured at 95°C for 5 min. Each 30 µg sample was applied to the gel well and 10 µg protein as mass marker (Precision Plus Dual Color, Bio-Rad, Hercules, CA, USA). Electrophoresis was run for 5 h in 150 V with cooling.

### 2.5. Silver Staining

Silver staining was performed according to the Shevchenko method [17], which is suitable for protein identification with mass spectrometry. All reagents were freshly prepared. Polyacrylamide gel after SDS-PAGE electrophoresis was fixed in the solution of 5% acetic acid and 50% methanol for 1 h. Next, it was washed in 50% methanol and MQ water, incubated for 3 min in 0.02% sodium thiosulphate, washed in MQ water, incubated in 0.15% silver nitrate and again washed with MQ water. The color reaction was developed by adding 2% sodium carbonate in 0.04% formaldehyde. The reaction was stopped with 5% acetic acid. Protein bands were cut directly after gel staining to avoid any contamination. Gel was documented with Gel Doc™ (Bio-Rad, Hercules, CA, USA).

### 2.6. Western Blotting

The immunoreactivity of isolated proteins was evaluated by Western blotting [18]. After the electrophoresis the gel was incubated in the transfer buffer (10 mM Tris-HCl, 150 mM glycine, 20% methanol, pH 8.3) for 30 min. Then, proteins from the gel were transferred to the polyvinylidene difluoride Immobilon P membrane (Merck, Darmstadt, Germany), using Trans Blot^®^ SD Semi-Dry Transfer Cell (Bio-Rad, Hercules, CA, USA), 1 h, 25 V. The membrane was then blocked with 1% BSA in TBS-T buffer (Tris Buffered Saline, 20 mM Tris, pH 7.5, 150 mM NaCl, 0.1% Tween20^®^), for 1 h at RT, with shaking. The primary antibodies (patients’ sera or umbilical cord blood sera) were added to the membrane in the dilution of 1:100 in TBS-T buffer with the addition of 0.1% BSA, incubated overnight at 4 °C. Then, the membrane was washed three times for 10 min in TBS-T. Subsequently, the secondary anti-human IgG antibodies conjugated with alkaline phosphatase (cat. A1543-1ml, Sigma-Aldrich, Saint Louis, MO, USA), were added in the dilution of 1:10,000 in TBS-T buffer, and incubated for 1 h at RT with shaking. The membrane was washed as previously described, and the color reaction was developed by adding NBT/BCIP solution (Sigma-Aldrich, Saint Louis, MO, USA). The reaction was stopped by multiple washing in water. The membrane was documented with Gel Doc™ (Bio-Rad, Hercules, CA, USA).

### 2.7. Protein Identification

The protein profiles visualized on the gel and blot were compared and immunoreactive proteins were cut from the gel. Then they were digested with trypsin, resulting peptides were separated by liquid chromatography and identified by LC-MS-MS/MS Orbitrap (Thermo Fisher Scientific, Waltham, MA, USA). The obtained peptide masses were compared with those collected in National Center for Biotechnology Information (NCBI) and UniProt databases by using MASCOT (Matrix Science Inc, Boston, MA, USA http://www.matrixscience.com/). All searches were done looking for proteins from the *Peptoclostridium difficile*.

### 2.8. Analysis of Sequence Homology

Protein amino acid sequence was evaluated in the terms of its preservation between CD strains as well as its homology to other bacterial strains. The analysis was performed using the basic local alignment search tool (BLAST, https://blast.ncbi.nlm.nih.gov/Blast.cgi) on the NCBI server [19].

### 2.9. Protein Modeling

It was not possible to build a 3D model for the identified protein since it has no crystalized homologs which could serve as a base. Thus the analysis of the amino acid sequence was performed basing on the identification of conserved domains (BLAST) and the secondary structure modelling using PredictProtein server (Technical University of Munich, https://www.predictprotein.org/) [20]. The input sequence of Cwp22 protein was WP_009890957.

### 2.10. Predicting Epitopes

The linear epitopes for B lymphocytes were predicted using two bioinformatic tools, BEPIPred server (Technical University of Denmark, http://www.cbs.dtu.dk/services/BepiPred/) [21], and BCPred server (Pennsylvania State University, http://ailab.ist.psu.edu/bcpred/) [22]. These tools use different algorithms for the search. The input sequence of Cwp22 protein was WP_009890957. Linear epitopes for T lymphocytes were predicted using TepiTool (Immune Epitope Database and Analysis Resource, IEDB, http://tools.iedb.org/tepitool/). The base was searched considering 27 most frequent MHC alleles (projected by the program). The input sequence of Cwp22 protein was WP_009890957 Results of both predictions and the protein structural model (exposed and functional regions) were used for designing amino acid sequences of potential epitopes. The design was mostly focused on targeting the functional parts of the proteins.

### 2.11. Chemical Synthesis of Peptides

The peptides were synthesized using PEPSCAN procedure [23] with some modifications. Peptides were synthesized on plastic pins, NCP type (MIA 10750001, Mimotopes, Mulgrave, Australia). F-moc amino acids with blocked side groups were used (Mimotopes, Mulgrave, Australia). In the first step, plastic pins were deprotected in 20% piperidine in dimethylformamide (DMF) for 1 h in a sealed vessel. Then, pins were washed and coupled with amino acid using the DMF solution of 60 mM amino acid, 65 mM 1-hydroxybenzotriazole and 60 mM diisopropylcarbodiimide. The reaction was monitored by 0.5 mM bromophenol blue—the decoloring of the solution implicates the reaction finishing (usually after 4 h). Coupling reaction was performed at RT in sealed vessel. Pins were washed, dried and incubated in DMF and conjugated with next amino acid. The reaction was repeated until the whole amino acid sequence was obtained. Finally, peptides side groups were deprotected with 2.5% anisole, 2.5% 1,2-dithioethane in trifluoroacetic acid. After the final wash in 0.5% acetic acid in 50% methanol the pins were dried and stored at −20 °C until further use.

### 2.12. Pin Enzyme-Linked Immunosorbent Assay

The immunoreactivity of pin-bound peptides was evaluated by enzyme-linked immunosorbent assay (ELISA) [24]. In this experiment three types of sera were used: acute CDI patients, umbilical cord blood sera and normal sera. First, pins were balanced in TBS-T for 10 min and blocked in 1% BSA in TBS-T for 1 h at RT with shaking. Pooled sera of above groups were used as primary antibodies in the dilution of 1:1000 in TBS-T. Pins were incubated in diluted sera for 2 h at RT with shaking. Secondary antibodies used were anti-human IgG conjugated with alkaline phosphatase, in dilution of 1:10,000 in TBS-T. Pins were incubated with secondary antibodies for 1 h at RT. The color reaction was developed using Alkaline Phosphatase Yellow Liquid Substrate System (cat. P7998, Sigma-Aldrich, Saint Louis, MO, USA) and the absorbance was measured in 405 nm (PowerWave HT, BioTek Instruments, Vermont, VT, USA). Each assay was repeated at least three times. Peptide-bound antibodies were removed from the peptides by 10 min disruption performed in the solution of 1% SDS, 0.1% β-mercaptoethanol, 0.1 M Na_3_PO_4_, pH 7.2 preheated to 60 °C. Pins were placed in the disruption buffer and sonicated in water bath sonicator (7 kW/25 kHz). Next, pins were washed in MQ water and methanol, dried, stored at −20 °C.

### 2.13. Epitope Identification

Immunoreactive 16-amino acid peptides were further analyzed—shortened peptides were synthesized from the C- or N-end. The immunoreactivity of the shortened sequences was tested. Amino acids essential for the immunoreactivity were then identified by the substitution of each amino acid with alanine, glycine or valine.

### 2.14. Epitope Analysis

Following, obtained sequences were checked for their cross-reactivity and autoimmunoreactivity. The IEDB database (https://www.iedb.org/) was searched for checking if identified epitopes are already identified [25]. The search was unrestricted to organism, nor MHC type, sequences of at least 70% similarity were checked. Also, the database of human autoepitopes (AAgAtlas database 1.0, http://biokb.ncpsb.org/aagatlas) was searched [26].

## 3. Results

### 3.1. The Identification of Cwp22 as a New Immunoreactive CD Surface Protein

**Figure 1:**
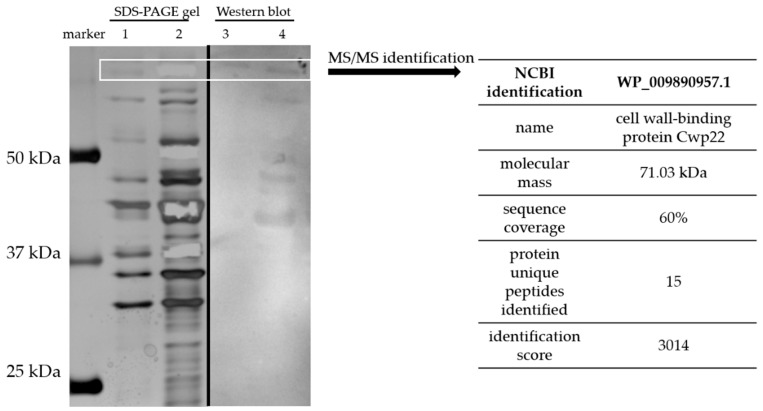
Identification of Cwp22 protein as a new immunoreactive protein from *Clostridioides difficile*. Surface proteins were isolated using 1 M LiCl, analyzed by SDS-PAGE electrophoresis (lanes 1 and 2) and western blotting (lanes 3 and 4). Lane 1—surface protein profile of R20291; lane 2—surface protein profile of CD20; lane 3—blotting result of R20291; lane 4—blotting result of CD20. White box indicates bands selected for identification by mass spectrometry. Results of the identification are shown in the table on the right.

Surface proteins were isolated using gentle washing off method without disruption of the bacterial cell [15]. Surface proteins were washed off using 1 M LiCl and the resulting protein solution was separated using two 1D SDS-PAGE electrophoresis gels. Next, one of the gels was subjected to Western blotting and tested with CDI patient blood serum. Second gel was stained with silver method [17]. Immunoreactive protein bands were excised from the SDS-PAGE gel immediately after staining and identified using mass spectrometry. The electrophoretic profile of surface proteins isolated from CD clinical strain (CD20, deposited as PCM2826) was similar to those previously published [27], although far more abundant in bands than a reference strain R20291 (Figure 1). The result of mass spectrometry of tryptic peptide fragments and MASCOT analysis showed that the immunoreactive protein of the molecular mass about 70 kDa is Cwp22 (Figure 1), one of the surface proteins of CD. The identification score was 3014. Fifteen unique peptides were identified during the analysis which allowed for reliable identification.

### 3.2. In Silico Analysis Shows that Cwp22 Protein is a Good Vaccine Antigen Candidate

The *cwp22* gene is conserved and present in various CD strains [28]. The bioinformatic analysis of Cwp22 amino acid sequence performed by BLAST showed that the protein is preserved in many CD strains. The analysis was performed using all 92 available Cwp22 protein sequences. Table 1 summarizes results for 22 strains for which it was possible to assign precise strain name. Bacteria present in the NCBI database were isolated from different sources: adults, children, acute CDI patients and asymptomatic carriers. Analyzed strains had different toxin profiles, however we were unable to fully characterize all strains used in this comparison (there were no data in the database). Gathered sequences were compared with Cwp22 protein sequence WP_009890957. The analysis showed that Cwp22 is a highly conserved protein. The percentage of identical amino acids in Cwp22 protein from different CD strains is at the range 91.93–99.84.

Since high homology of the vaccine antigen to proteins from other organisms may cause cross-reactivity we analyzed Cwp22 protein homology using BLAST algorithm. The sequence of Cwp22 protein is moderately homological to other l,d-transpeptidases, some hypothetical proteins and a surface protein from strains with questionable clinical significance (Table 2), mostly closely related to the *Clostridioides* genus. The percentage of identical amino acids is between 41.90 to 56.07. The highest similarity is in the N-end region of the protein (data now shown).

**Figure 2:**
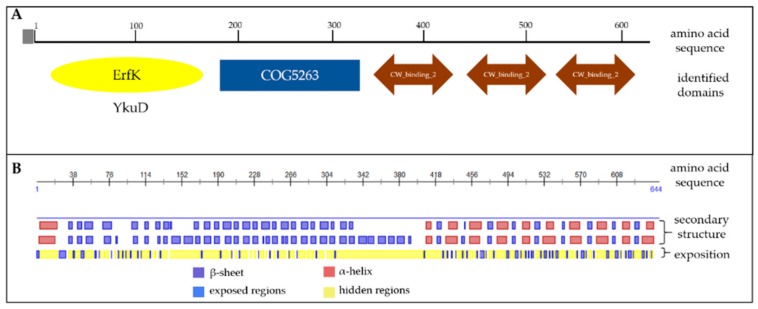
The analysis of the conserved domains (**A**) and secondary structure (**B**) of Cwp22 protein. Conserved domains were identified using BLAST algorithm. The secondary structure was predicted by PredictProtein server.

Based on the amino acid sequence we studied the presence of conserved domains in the protein using BLAST algorithm. Aiming functional regions of the protein may have an influence on vaccine efficacy. The analysis identified a YkuD domain possessing the activity of l,d-transpeptidase and playing the role in producing bacterial peptidoglycan [29] (Figure 2A). On the other end of the Cwp22 protein sequence there are three CW_binding_2 domains which are responsible for binding this protein to the bacteria cell wall. The protein sequence is preceded by a signal sequence directing the protein outside the bacteria cell [30]. This prediction suggests that the functional part of the protein is surface-exposed.

It was not possible to build a reliable protein model because there is no crystallized protein having enough homology to the Cwp22 protein that could serve as templates for the prediction. We used the mathematical model to predict secondary structure of Cwp22 (PredictProtein server) (Figure 2B). The analysis showed the presence of many α-helix structures localized in the region which binds the protein to the cell wall. The functional part of the protein is mostly built of β-sheets and loops.

These data indicate Cwp22 protein as a conserved protein in CD strains but having no significant homology to proteins from other strains and confirm that Cwp22 protein is a promising target for further vaccine research.

### 3.3. Predicting the Epitopes of Cwp22 Protein

**Figure 3:**
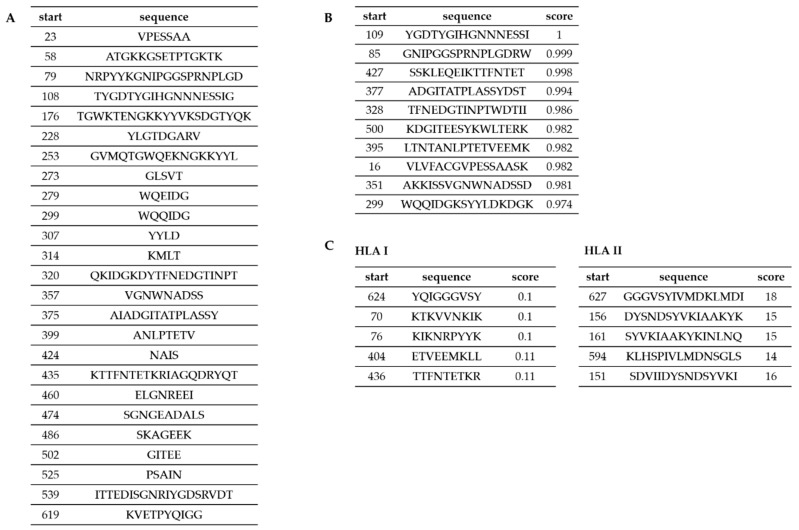
In silico analysis of Cwp22 protein epitopes. **A** shows B-lymphocyte epitopes identified by BEPIPred server; **B** shows B-lymphocyte epitopes identified by BCPred server; **C** shows T-lymphocytes epitopes identified by TepiTool server.

**Figure 4:**
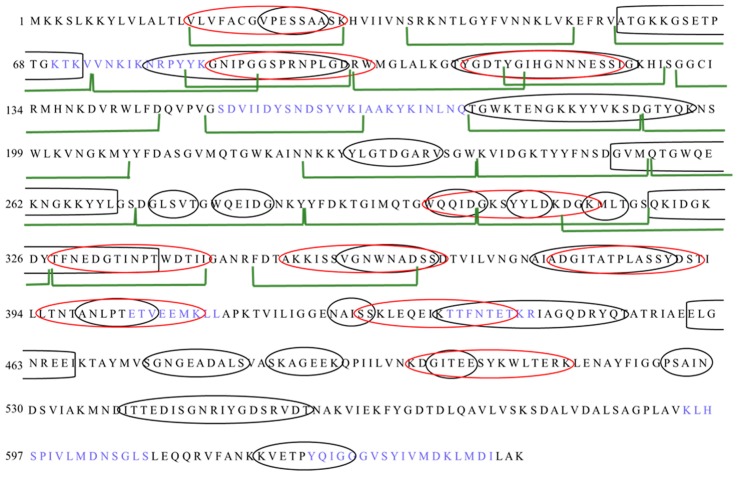
The strategy of designing 16-amino acid peptides for chemical synthesis. Black ellipses indicate sequences identified by BEPIPred. Red ellipses indicate sequences identified by BCPred. Blue letters indicate sequences identified by TepiTool. Green markers indicate sequences selected for synthesis.

Data obtained by domain identification and protein modeling were used in the process of designing peptides for epitope mapping. Additionally, we performed a set of epitope predictions using bioinformatics tools in order to design peptides covering possible T- and B-cell epitopes. We applied two prediction servers (BCPred, BEPIPred) for prediction of B-lymphocyte epitopes and TepiTool server for prediction of T-lymphocyte epitopes. Predictions of the epitopes for B-lymphocytes performed using two different servers (BCPred and BEPIPred) gave complementary results (Figure 3A,B). The epitopes proposed for T-lymphocytes were mostly different than those proposed for B-lymphocytes. These proposed sequences were considered when designing peptides for chemical synthesis (Figure 4). During the peptide selection process we focused on the first part of the protein which is responsible for its functionality since it contains the YkuD domian.

### 3.4. Epitope Mapping

**Figure 5:**
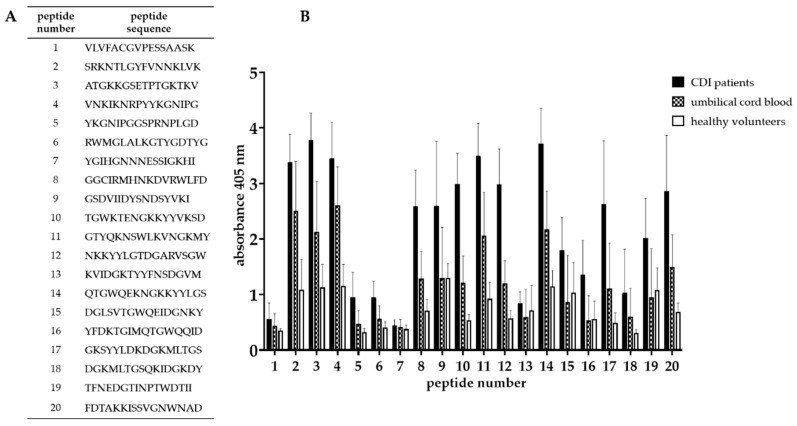
Epitope mapping of Cwp22 protein. (**A**)—peptide sequences synthesized based on in silico analysis; (**B**)—result of epitope mapping by ELISA using sera from three groups. ELISA was performed using pin-bound peptides and 96-well plate. Primary antibody was detected by anti-human IgG-AP conjugated goat antibody which reacts with AP Yellow substrate. The result of colorimetric reaction was measured by reading absorbance in 405 nm. The figure shows results from at least three independent measurements, means with SD.

In order to determine the amino acid sequence recognized by antibodies, the truncation peptide library was prepared using the PEPSCAN method. The epitope mapping begun with assessment of the immunoreactivity of chemically synthesized peptides which were designed as is shown in Figure 4. The twenty-one peptides (16-amino acid long) corresponding to predicted epitopes were synthesized (Figure 5A). We tested their immunoreactivity against pooled sera from three groups: group of patients undergoing CDI (*n* = 15), group of umbilical cord blood sera (*n* = 10), group of healthy volunteers (*n* = 10). The immunoreactivity profile is similar for all groups, however different titers of immunoreactivity were observed (Figure 5B). The highest level of immunoreactivity was in the CDI patients’ group, next, the umbilical cord blood sera group and the last healthy volunteers. The highest titers of immunoreactivity were found for peptides 2, 3, 4, 11, 14, 17, and 20, whereas peptides with the lowest titer of immunoreactivity were 1, 5, 6, 7, 13, 16, and 18. The variance in immunoreactivity was statistically significant (ANOVA, *p* < 0.05) and peptides with the lowest titer of immunoreactivity were removed from further testing.

**Figure 6:**
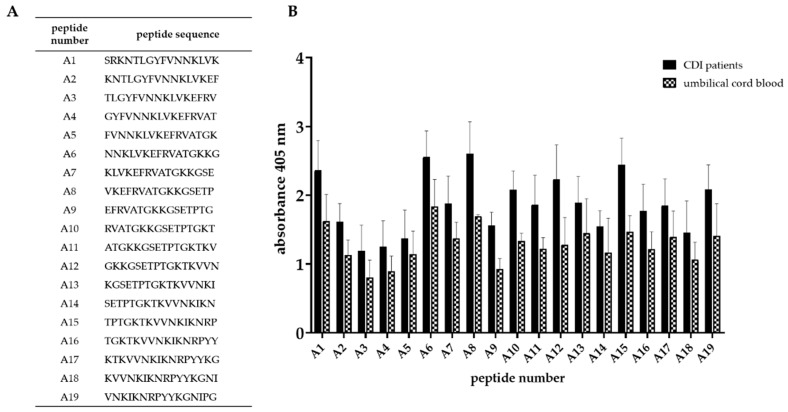
Epitope mapping of highly immunoreactive region of Cwp22 protein. **A**—peptide sequences designed for epitope mapping; **B**—the result of epitope mapping by ELISA using sera from two groups. ELISA was performed using pin-bound peptides and 96-well plate. Primary antibody was detected by anti-human IgG-AP conjugated goat antibody which reacts with AP Yellow substrate. The result of colorimetric reaction was measured by reading absorbance in 405 nm. The figure shows results from at least three independent measurements, means with SD.

We identified a region of the strongest immunoreactivity (for all three tested groups) that included sequences from peptides 2 to 4 and two most immunoreactive sequences 11 and 14 (Figure 5B). Amino acid sequence covering peptides from 2 to 4 falls into the YkuD functional domain, so we decided to map this region in further details. The two additional highly immunoreactive sequences A6 and A8 located within YkuD functional domain (Figure 6) were identified by applying modified overlapping peptide libraries. We decided to further investigate peptide sequence covering sequences A6–A8, namely NNKLVKEFRVATGKKGSETP.

To sum up, peptides selected for further minimal epitope mapping are peptides A6–A8 (^48^NNKLVKEFRVATGKKGSETP^67^), 11 (^192^GTYQKNSWLKVNGKMY^207^) and 14 (^265^QTGWQEKNGKKYYLGS^280^).

### 3.5. Defining the Epitopes of Cwp22 Protein

**Figure 7:**
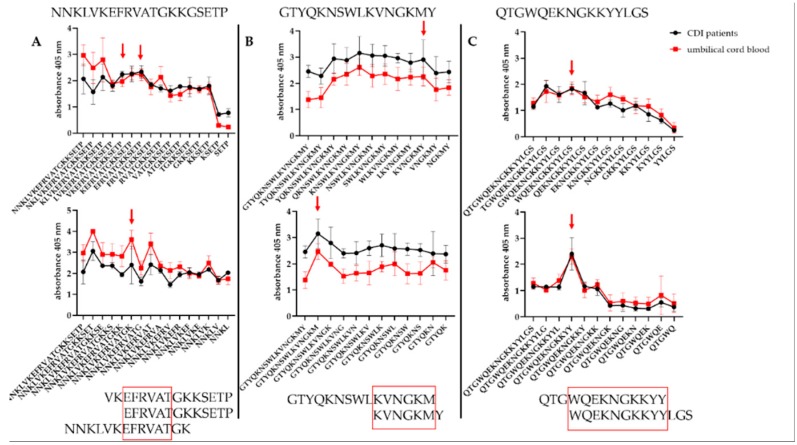
Finding epitopes in Cwp22 protein. **A** shows the shortening of NNKLVKEFRVATGKKGSETP peptide; **B** shows the shortening of GTYQKNSWLKVNGKMY peptide; **C** shows the shortening of QTGWQEKNGKKYYLGS peptide. Peptides were shortened either from C- (upper panel) or N- (lower panel) end and tested for their immunoreactivity by pin-bound ELISA. Experiment was repeated three times. Red arrows indicate the shortest, still immunoreactive sequences.

Peptides A6–A8, 11 and 14 were shortened from N- or C- ends in order to define the shortest, but still strongly immunoreactive amino acid sequence. Two kinds of sera were used obtained from CDI patients and umbilical cord blood. During the analysis of immunoreactivity, we compared the shortest yet immunoreactive peptides (Figure 7). In the A6–A8 (NNKLVKEFRVATGKKGSETP) peptide we identified ^54^EFRVAT^59^ epitope since the removal of its C-end glutamic acid or N-end threonine caused a decrease in immunoreactivity. We noticed that in the peptide number 11 (GTYQKNSWLKVNGKMY) after removing the second lysine from C-end or methionine from N-end resulted in the substantial loss of peptide immunoreactivity which indicates ^201^KVNGKM^206^ as the epitope. In the peptide number 14 (QTGWQEKNGKKYYLGS) removing tryptophan from the C-end or tyrosine from the N-end resulted in the loss of immunoreactivity indicating ^268^WQEKNGKKYY^277^ as the epitope.

To sum up, we found the shortest immunoreactive peptides for each tested sequence (A6–A8, 11 and 14) (Figure 7). Epitope ^268^WQEKNGKKYY^277^ located in sequence 14 is a part of probable B-cell epitope (Figure 3). Mostly interesting is that sequence A6–A8 showed higher immunoreactivity titer tested with the sera from umbilical cord blood than from CDI patients’ sera. Such finding suggests that these sequences possess potential protective activity.

### 3.6. Epitopes Characteristics

We characterized physicochemical properties of studied epitopes, completed their autoimmunoreactivity and crossreactivity by in silico analysis. All three identified epitopes are soluble in water. ^54^EFRVAT^59^ and ^201^KVNGKM^206^ form loops. ^268^WQEKNGKKYY^277^ forms an alpha helix. The three epitopes differ in their pI, ^54^EFRVAT^59^ has no charge in physiological pH, ^201^KVNGKM^206^ and ^268^WQEKNGKKYY^277^ have the same charge in physiological pH which is 2 (Table 3).

The potential cross-reactivity was analyzed by comparing homological sequences present in the NCBI database and the analysis was performed by BLAST algorithm. The sequence ^54^EFRVAT^59^ was found to be quite common since it is found in sequences of 130 bacteria strains, however mostly in hypothetical proteins. Also, it is present in cellulose biosynthesis protein from *Burkholderia multivorans*, hyaluronoglucosaminidase of *Streptomyces* sp. *2131.1* or vitamin B12-dependent ribonucleotide reductase of *Rhizobiales bacterium*. The sequence ^201^KVNGKM^206^ was found in many different species like *Plasmodium malariae* (erythrocyte membrane-associated antigen), *Fusobacterium varium* (autotransporter domain-containing protein). It was also discovered in bifunctional (p)ppGpp synthetase/guanosine-3′,5′-bis(diphosphate) 3′-pyrophosphohydrolase from some *Lactobacilli* and *Streptococcus*, yet these strains are colonizing mostly animals. Probably the frequent occurrence of this sequence it is connected with the epitope location in a conserved domain COG5263, which is a choline binding sequence [31]. The sequence ^268^WQEKNGKKYY^277^ is specific only for *Clostridioides*.

For the potential autoimmunoreactivity we checked an IEDB database of known autoepitopes. The search was not restricted to any host nor MHC type. The result of the search for ^54^EFRVAT^59^ showed that there is a congenial epitope in a staphylokinase produced by *Staphylococcus aureus* (TAYKEFRVAEADPSAKI). Moreover, T-lymphocytes specific for this epitope (TAYKEFRVAEADPSAKI) were found in human [32]. There are no known epitopes or autoepitopes for ^201^KVNGKM^206^ nor ^268^WQEKNGKKYY^277^.

## 4. Discussion

In this study, we identified a new immunoreactive surface protein isolated from CD which is named Cwp22 and we mapped its epitopes using human sera obtained from CDI patient sera and umbilical cord blood sera. We defined three specific epitopes: ^54^EFRVAT^59^, ^201^KVNGKM^206^, and ^268^WQEKNGKKYY^277^, that we characterized thoroughly and evaluated for future use in a vaccine construction.

Peripheral CDI patients blood sera and umbilical cord blood sera were used for Cwp22 protein epitope mapping. The antibody transfer through the placenta is an active and selective process since most molecules of such high molecular mass do not cross the placenta. Those are mostly protein-specific IgG antibodies which protect the baby after the birth [33]. In case of some pathogen specific antibodies are even selectively accumulated in the fetus [34]. Based on the above we routinely use umbilical cord blood sera for mapping bacterial proteins since maternal antibodies passed to the fetus may have highly protective properties [14,24]. We observed, that anti-Cwp22 protein antibodies from umbilical cord blood sera are more immunoreactive with Cwp22-derived peptides than those sera gathered from healthy volunteer group (Figure 5), in some cases they almost reach the activity of antibodies from CDI patient group (Figure 6) or even are more active (Figure 7A). It is one of the limitations of the study not knowing if mothers were asymptomatic carriers of CD or if they did have contact with these bacteria since there is no routine testing for CD in healthy people. Nevertheless, this enrichment of specific antibodies against Cwp22 protein in umbilical cord blood sera comparing to healthy volunteers’ sera may be a proof of their great importance in protective immunity against CD.

When we started looking for new anti-CD vaccine antigens, the Cwp22 protein was identified bioinformatically as one of the cell wall proteins [35]. Next, it was found in the CD proteome [36], characterized [29] and recently, predicted to be a good vaccine antigen by Zhou et al. [13]. Now, we are the first to identify Cwp22 protein as an immunoreactive one using CDI patient sera. There are some papers concerning searching for the immunoreactive surface proteins of CD [27,37,38]. Wright, et al. [27] extracted surface proteins from CD M9 (ribotype 017, toxin type A^+^, B^-^) and separated them using 2D electrophoresis. Then the gels were subjected to Western blotting with CDI patient sera followed by mass spectroscopy identification. These authors identified forty-two different immunoreactive proteins, but no Cwp22 protein among them. This can be explained using different extraction method. Wright, et al. used TS buffer (10 mM Tris/HCl (pH 6.9), 10 mM MgCl2, 0.5 M sucrose) with the addition of mutanolysin, lysozyme, lysostaphin, RNase and 4-(2-aminoethyl)benzenesulfonyl fluoride hydrochloride which mostly extracted proteins of pI between 4 and 6, whereas Cwp22 protein pI is 8.89 [13].

Cwp22 protein is an enzyme that is involved in production of unusual peptidoglycan in CD since it mainly contains 3→3 crosslinks [15], which occurs prevalently in mycobacteria [39]. There are three paralogs of this enzyme in CD named Ldt_Cd1_, Ldt_Cd2_, Ldt_Cd3_ (l,d-transpeptidase, Ldt) [40]. Deletion of Ldt_Cd1_ and Ldt_Cd2_ led to decreased content of 3→3 peptidoglycan crosslinks. So far, it was not possible to inactivate Ldt_Cd3_ by deletion resulting in no CD mutant totally lacking 3→3 peptidoglycan crosslinks. The absence of such a mutant makes it difficult to study the biological meaning of 3→3 peptidoglycan crosslinks. About 30% of CD peptidoglycan has the 4→3 crosslinks which are typical for bacteria and are produced by D, d-transpeptidases (PBP). The activity of PBPs is arrested by all classes of β-lactams whereas Ldts can be inhibited by carbapenems only. In *E. faecium* changing PBPs to Ldts ensures high level resistance to β-lactams of the penam class, for example MIC for penicillin can be >2000 µg/mL [41]. We are still lacking data about similar phenomenon in CD but aiming Cwp22 protein could become at least helpful in the process of antibiotic treatment.

Cwp22 protein plays a pleiotropic role in CD. ClosTron-generated *cwp22* mutant was characterized [13]. Zhu, et al. showed *cwp22* CD mutant as less viable, producing lower levels of TcdA and TcdB toxins during early stage of growth and autolyzing faster than the parent strain. More surprisingly, is that the *cwp22* mutant showed impaired adherence in vegetative strain and spores. In vivo studies on mice showed impaired colonization and pathogenesis when they were infected with *cwp22* mutant strain as to compare with parent strain. This means that targeting Cwp22 protein, for example with therapeutic antibodies, can have tremendous impact on the CD virulence.

During the pathogen invasion human body builds up humoral response producing a substantial variety of antibodies with different specificities. Not all of them are protective. Only a portion of these antibodies targets crucial entities for the pathogen virulence. In this paper, we report for the first time a surface protein Cwp22 as an immunoreactive one. Previously, it has been shown that a peptide was more immunogenic than the intact protein as shown in higher antibodies titer induced [42]. Wherefore, we mapped the epitopes of Cwp22 protein by combining bioinformatics approach and empirical study. Potentially immunoreactive peptides were designed by taking into account B-cell and T-cell epitope predictions and modelled secondary structure. Further, epitope mapping by PEPSCAN method [23] exposed three epitopes ^54^EFRVAT^59^, ^201^KVNGKM^206^ and ^268^WQEKNGKKYY^277^. A particularly interesting epitope, the ^54^EFRVAT^59^ is located in the domain responsible for the biological activity of the enzyme. Its structure was bioinformatically predicted to be a coil (Table 3). In native protein it located on a β-sheet. The second epitope ^201^KVNGKM^206^ is also predicted to form a coil. The third epitope was predicted to be an alpha helix (Table 3). In native protein it was predicted to be located on an exposed loop. Predicted secondary structure showed that the part of protein containing all three identified epitopes forms mostly β-sheets and coils, therefore it should be further evaluated whether this change in structure influences peptide immunoreactivity. Our epitopes have six to ten amino acids which is in line with the generally accepted principle that reactive linear epitopes should have five to twenty-two amino acid residues [43] or six to nine polar amino acids [44]. Recently, Pyclik, et al. published data showing a very short 4 amino acid sequence to be strongly immunoreactive [45]. However, it was suggested previously that 15 amino acid sequence are optimal immunogens [46]. In further studies, it might become necessary to add flanking amino acids to increase immunogenicity of identified epitopes. Further studies concerning protective properties of all identified epitopes are in progress.

Vaccines based on proteins and peptides should be carefully tested for cross-reactions. Too much similarity of vaccine antigen sequence to proteins of own microbiota or self-antigens can lead to unpredictable effects. As we previously showed some bacterial conservative antigens, like glyceraldehyde 3-phosphate dehydrogenase, can be implicated in autoimmunoreactivity [14]. There are known T-cell autoepitopes that cross-react with M-protein group A streptococci which was previously suggested as a vaccine candidate [47]. Cwp22 protein amino acid sequence is highly conserved among CD strains regardless of their ribotype or type of produced toxins (Table 1) and have low homology to other l,d-transpeptidases (Table 2). We searched for the epitopes’ sequences (^54^EFRVAT^59^, ^201^KVNGKM^206^, ^268^WQEKNGKKYY^277^) in IEDB database and they did not match with any known autoepitope.

There is a possibility of host microbiota interfering with vaccine. Williams, et al. induced anti- HIV-1 env response by vaccination of healthy participants. They found that even though the vaccine contained both gp120 and gp41 the response was focused on gp41. The antibodies were mostly non-neutralizing and cross-reactive with intestinal microbiota [48]. In order to avoid above effect, we examined epitope sequences homology to microbiota proteins using BLAST algorithm. ^54^EFRVAT^59^, ^201^KVNGKM^206^ were found in some bacteria with uncertain clinical importance. Nevertheless, this finding should be further evaluated. Also, we tested weather epitopes determined in this study occur in human proteins. Epitopes ^54^EFRVAT^59^, ^201^KVNGKM^206^, and ^268^WQEKNGKKYY^277^ were not found by us in human proteins sequences deposited in NCBI database.

In conclusion, we documented that Cwp22 protein is one of the immunoreactive proteins present on the surface of *C. difficile*. We mapped its epitopes using CDI patient blood and umbilical cord blood sera, analyzed their homology to proteins from other bacteria strains, also human proteins. We comprehensively characterized the epitopes using bioinformatics and empirical methods. Based on our studies, the epitopes ^54^EFRVAT^59^, ^201^KVNGKM^206^, and ^268^WQEKNGKKYY^277^ are an attractive vaccine antigen candidates.

## Figures and Tables

**Table 1:** Conservation of the Cwp22 protein amino acid sequence between *Clostridioides difficile* strains. Data collected from NCBI and BLAST servers.

NCBI Identification	CD Strain	Characteristics	Sequence Coverage	% of Identical Amino Acids
CBA64897.1	CD196	Isolated from an adult, ribotype 027, producing toxins	100%	99.84
PBF93815.1	7468-NonSp/ST97	Isolated from a child, producing toxins	100%	99.69
EQG56151.1	DA00145		100%	99.69
EQH98359.1	F314	Acute CDI, producing toxins	100%	99.69
OFU21376.1	HMSC19C09		100%	99.69
OFU05949.1	HMSC19D07		100%	99.53
EHJ35368.1	70-100-2010		100%	99.38
PBG98735.1	7481-NonSp/ST59		100%	99.22
EQF50178.1	CD174	Asymptomatic carriage, no toxins	100%	99.07
EQE30253.1	CD34	Asymptomatic carriage, no toxins	100%	99.22
AFV69537.1	C192	Ribotype 012, hipervirulent	100%	99.22
EQJ05755.1	P7	Reference strain, producing toxins	100%	99.07
PBE99335.1	5573-CF/ST37	Producing TcdB	100%	98.76
EQE84199.1	CD69	Acute CDI, producing toxins	100%	98.91
OJT75205.1	NT422	Ribotype 231	98%	99.37
EFH06251.1	NAP08	Hipervirulent	100%	96.89
PBI20744	6615-NonSp/ST11	Isolated from a child, hipervirulent	100%	96.74
KPI50551	RA09-70	Producing TcdA	100%	93.17
PBE36357.1	5537-D/ST9	Isolated from a child, producing toxins	100%	91.93
ERM37167	P64	Asymptomatic carriage, producing toxins	79%	99.41
EQE47488	CD42	Acute CDI, producing toxins	73%	99.30
EQK74493	CD113	Relapse, producing toxins	72%	99.51

**Table 2:** The search results for proteins homological to CD Cwp22 protein. Data collected from NCBI database and analyzed by basic local alignment search tool (BLAST) algorithm.

NCBI Identification	Protein Name	Bacteria Strain	Sequence Coverage	% of Identical Aminoacids
WP_092726434.1	l,d-transpeptidase	*Romboutsia lituseburensis*	53%	56.07
WP_007287442.1	l,d-transpeptidase	*Clostridium bartletti*	53%	51.59
WP_084157842.1	Hipothetical protein	*Bacillus manliponensis*	58%	47.59
WP_082007829.1	Hipothetical protein	*Terrisporobacter othiniensis*	54%	52.10
WP_052404759.1	l,d-transpeptidase	*Bacillus rubiinfantis*	55%	48.66
WP_066389794.1	l,d-transpeptidase	*Bacillus mesonae*	52%	41.90
KLR55379.1	Surface protein	*Clostridium sordelli*	53%	45.98

**Table 3:** Epitope characteristics. The physicochemical properties were calculated by PepCalc. The secondary structure was modeled by PepFOLD server.

	^54^EFRVAT^59^	^201^KVNGKM^207^	^268^WQEKNGKKYY^277^
Molecular mass (g/mol)	721.8	675.84	1343.49
Water solubility	good	good	good
pI	6.86	10.69	9.9
Charge in physiological pH	0	+2	+2
Peptide structure